# Peptides from Natural or Rationally Designed Sources Can Be Used in Overweight, Obesity, and Type 2 Diabetes Therapies

**DOI:** 10.3390/molecules25051093

**Published:** 2020-02-29

**Authors:** Mayara C. F. Gewehr, Renata Silverio, José Cesar Rosa-Neto, Fabio S. Lira, Patrícia Reckziegel, Emer S. Ferro

**Affiliations:** 1 Department of Pharmacology, Biomedical Sciences Institute, University of São Paulo (USP), São Paulo 05508-000, Brazil; ferrari.mayaracalegaro@gmail.com; 2 Department of Pharmacology, Center of Biological Sciences, Federal University of Santa Catarina (UFSC), Florianópolis 88040-900, Brazil; resilveriodeluca@gmail.com; 3 Department of Cell and Developmental Biology, Biomedical Sciences Institute, University of São Paulo (USP), São Paulo 05508-000, Brazil; josecesar23@hotmail.com; 4 Department of Physical Education, São Paulo State University (UNESP), Presidente Prudente 19060-900, Brazil; fabioslira@gmail.com; 5 Department of Pharmacology, National Institute of Pharmacology and Molecular Biology (INFAR), Federal University of São Paulo (UNIFESP), São Paulo 05508-000, Brazil; reckziegel.patricia@gmail.com

**Keywords:** intracellular peptides, hemopressin, Pep19, overweight, obesity, type 2 diabetes

## Abstract

Overweight and obesity are among the most prominent health problems in the modern world, mostly because they are either associated with or increase the risk of other diseases such as type 2 diabetes, hypertension, and/or cancer. Most professional organizations define overweight and obesity according to individual body–mass index (BMI, weight in kilograms divided by height squared in meters). Overweight is defined as individuals with BMI from 25 to 29, and obesity as individuals with BMI ≥30. Obesity is the result of genetic, behavioral, environmental, physiological, social, and cultural factors that result in energy imbalance and promote excessive fat deposition. Despite all the knowledge concerning the pathophysiology of obesity, which is considered a disease, none of the existing treatments alone or in combination can normalize blood glucose concentration and prevent debilitating complications from obesity. This review discusses some new perspectives for overweight and obesity treatments, including the use of the new orally active cannabinoid peptide Pep19, the advantage of which is the absence of undesired central nervous system effects usually experienced with other cannabinoids.

## 1. Introduction

Overweight and obesity have multifactorial causes [1], including but not restricted to increased consumption of processed foods [2,3], which are usually rich in hydrogenated fats and simple carbohydrates and low in complex carbohydrates [4,5]. Mitochondrial mechanisms directly involved in cellular energy production are increasingly recognized as playing a role in the control of energy balance. A study of mitochondrial DNA identified genetic variants linked with excess adiposity and metabolic disturbances [6]. Emotional factors play an important role in the genesis of overweight and obesity. Longitudinal studies showed that depression, anxiety, and life stress increase the odds of weight gain with the development of overweight and obesity through multiple but incompletely established mechanisms [7]. The decline in energy expenditure is associated with a lack of regular physical activity [8].

Individual nutritional needs vary according to age, sex, health status, and physiological status, level of physical activity, and number of working hours. The energy balance is the result of the total energy (in calories) consumed and the total energy spent by the body during daily activities; when in energy balance, healthy adults do not gain or lose weight as the energy balance is zero. Calories are defined as the amount of energy (or heat) needed to increase the temperature of one gram of water by 1 °C; 1 calorie = 4.2 joules. In food, calories are usually represented in kcal. For example, for the energy content of food, proteins and carbohydrates provide 4 kcal/g, fats provide 9 kcal/g, and vitamins, minerals, and water do not provide energy. However, foods with similar carbohydrate contents do not necessarily have the same impact on blood glucose levels [9]. The concept of glycemic index (GI) classifies the blood-glucose-raising potential of carbohydrate foods relative to glucose or white bread [9]. Diets with a low GI may have beneficial effects on body weight and body composition and on certain risk factors in persons with overweight [10,11]. In a large European study, a modest increase in protein content and a modest reduction in the GI led to an improvement in the maintenance of weight loss [12].

The calculation of healthy weight is determined by the body–mass index (BMI), which is the person’s weight divided by the height squared of an individual. The World Health Organization (WHO) recommends a BMI between 18.5 and 25 for the population, avoiding weight gains greater than 5 kg in adult life [13]. Individuals with BMI index between 25 and 30 are considered overweight. For obesity, a BMI index ≥30 is considered a chronic disease for professional organizations [14]; the high relapse rates of individuals who are no longer obese are in agreement with considering obesity a chronic disease that requires long-term vigilance and weight management [1].

Like many mammals, humans show sexual differences in energy metabolism. Women have a higher proportion of body fat compared to men [15,16,17,18]. From the beginning of puberty until menopause, women maintain a higher percentage of fat mass than men, despite the lower energy intake per kilogram of lean mass [16]. In addition, women consume less kilojoules per kilogram of lean mass and burn fat more preferably during exercise compared to men [19]. During pregnancy, women store even greater amounts of fat that cannot be attributed solely to increased energy intake. These observations suggest that the relationship between kilojoules consumed and kilojoules used is different in men and women [15]. On average, men and women achieve an energy balance of 2400 and 2200 calories per day, respectively; an average of 2000 calories meets the energy needs of younger people (<15 years) [1]. The reason for these sexual differences in energy metabolism is not known; however, they may be related to sex steroids, differences in insulin resistance, or metabolic effects of other hormones, such as leptin [15]. Sex hormones potently control food intake and body weight [20,21]. Differences between sexes in the accumulation of adipose tissue in different deposits [22,23] significantly affect the metabolic phenotype, since adipokine production, insulin sensitivity, and the release of free fatty acid vary between deposits. Circulating plasma leptin levels are different between males and females; in humans, women increase circulating leptin levels for any degree of adiposity [20,24,25]. Leptin mRNA expression is increased in subcutaneous adipocytes compared with omental adipocytes, and the ratio of subcutaneous to omental leptin mRNA is higher in women than in men [26]. In addition, the metabolic rate per kilogram of adipose tissue is higher in women than in men [27]. Estrogen has an inhibitory effect on meal size and daily food intake, in addition to regulating daytime eating patterns [20,28]. Estrogen has direct effects on fat mass, since it regulates leptin expression in adipocytes and, to support these studies, leptin production was inhibited by androgens and promoted by estrogens [25,29,30]. Estrogen receptors were detected in the hypothalamic nuclei that control energy homeostasis, showing that central effects may also occur. Circulating estrogens bind to these receptors and alter the hypothalamic sensitivity to leptin-mediated signals, influencing leptin secretion and possibly metabolism and even fertility [31,32,33].

Therefore, obesity is a complex multifactorial disease. Here, peptides are in the center of this discussion, and are presented both as important physiological regulators of energy metabolism and therapeutic molecules that can efficiently help to control and treat overweight, obesity, and/or type 2 diabetes (T2D).

## 2. Brief Historical Perspective on Proteasome-Derived Intracellular Peptides: Possible Therapeutic Applications to Control and Treat Overweight, Obesity, and T2D

Cells have special mechanisms that break proteins and peptides into amino acids to be further used in de novo protein synthesis [34,35,36,37]. In the cell cytosol and nucleus, this process is mainly performed by the ubiquitin–proteasome system, followed by aminopeptidases [38] and oligopeptidases such as neurolysin (Nln) and thimet oligopeptidase (THOP1). The majority of the proteasomal products of a cell are thought to be rapidly broken down further into free amino acids [39]. However, some exceptions exist, such as proteasome-processed peptides that escape further degradation being presented on the cell surface as major histocompatibility class-I (MHC-I) antigens [38,40,41,42]. Hundreds of non-MHC-I antigenic peptides are stable within cells, coined *intracellular peptides* [43,44].

By definition, intracellular peptides are functional peptides formed by proteasome activity during regular degradation of intracellular proteins. Intracellular peptides tend to have a longer half-life than most non-antigenic proteasome-processed peptides; however, the reason these peptides last longer inside the cells remains uninvestigated [43]. A small group of intracellular peptides were found to be produced from short open reading frames and from defective ribosomes [45,46,47]. Intracellular peptide precursor proteins have major subcellular locations in the nucleus, cytosol, or mitochondria [48].

The seminal identification of intracellular peptides was based on a substrate-capture assay that employs catalytically inactive forms of either thimet oligopeptidase (EC3.4.24.15; EP24.15, THOP1) or neurolysin (EC3.4.24.16; Nln) to identify novel pharmacological active peptides such as rat hemopressin (HP, PVNFKFLSH) and AGHLDDLPGALSAL (AGH) [44,49,50]. More recently, identifying intracellular peptides from biological samples has been performed directly from tissue or cells homogenates [51], and the rapid progress in this area is allowing the identification of thousands of intracellular peptides that have been sequenced in plants, yeast, zebrafish, rodents, and human cells and tissues [52].

Intracellular peptides are able to modulate G-protein coupled receptors (GPCR) isoproterenol and angiotensin II signal transduction in HEK293 and Chinese hamster ovary (CHO) cells [50]. These intracellular peptides affecting GPCR signal transduction were isolated from rat brain homogenates using the inactive THOP1 substrate-capture assay [50]. These studies also demonstrated that the overexpression of THOP1, which has been shown to be involved in the metabolism of specific intracellular peptides [53] and modulation of signal transduction of angiotensin II (AT1) and β-adrenergic GPCR in CHO and HEK293 cells. Recent studies in HEK293 cells showed that most of these intracellular peptides are generated by the proteasome, where additional intracellular peptide-generating peptidases also exist [52,54,55,56].

Different treatments and/or diseases modify the relative concentration of specific intracellular peptides present inside the cells and tissues, suggesting pathophysiological functions [51,54,55]. In challenge conditions, cells start accumulating or losing specific intracellular peptides that are biologically functional in such conditions. For example, Wistar rats fed a Western diet developed obesity and insulin resistance and had a greater increase in the weight of epididymal, mesenteric, and retroperitoneal adipose tissues compared with rats fed a control diet [57]. The relative levels of intracellular peptides identified in the epididymal adipose tissue of rats fed a hypercaloric Western diet were compared with the levels of intracellular peptides identified in epididymal adipose tissue of rats fed a control diet using semiquantitative mass spectrometry [57]. Among the 10 peptides identified, two were slightly increased, TVGDVNTDRPGLLDL (DBI) and GDVNTDRPGLLDL (LDBI), both derived from acyl-CoA-binding protein [57]. At concentrations between 0.1 and 1 nM, both DBI and LDBI facilitated glucose transportation, stimulated by insulin, both in regular and insulin-resistant 3T3L1 differentiated adipocytes. DBI was shown to bind to heat shock protein 8 only in epididymal adipose tissue extracts obtained from rats fed a Western diet. LDBI, a shorter version of DBI binds to annexin A6, asporin, adenosine-triphosphate synthase (ATP synthase), H^+^ transporting mitochondrial F1 complex beta polypeptide isoform, CRA_a, complement component 4A, protein 1 (HMG-1), and Ig gamma-2A chain C region in addition to binding heat shock protein 8, but only in the epididymal adipose tissue extracts obtained from rats fed a Western diet [57].

Studies using transgenic animals for angiotensin-converting enzyme (ACE) containing one, two, or three copies of the *ACE* gene showed that under normal feeding conditions, animals with three copies of the *ACE* gene consume more food to maintain the same body weight [58]. However, when challenged with a high-fat diet, these animals consumed the same amount of food while having a lower body weight than animals containing one or two copies of the *ACE* gene [58]. Treatment with losartan, an AT1 receptor antagonist of angiotensin II, does not alter the metabolic profile described above for these animals, suggesting that independent mechanisms of the renin-angiotensin-ACE system are related to the phenotypic differences observed in these animals. Among the changes observed in these animals is a reduction in neurolysin activity in the adipose tissue of animals with three copies of the *ACE* gene compared with animals containing one copy of the *ACE* gene. Adipose tissue from animals containing three copies of the *ACE* gene had an intracellular peptide composition distinct from that of animals containing only one copy of that gene. Most intracellular peptides identified in animals containing one or three copies of the *ACE* gene have a potential phosphorylation site, and two of these peptides were shown to competitively inhibit the phosphorylation of a standard protein kinase C substrate. Thus, intracellular peptides were suggested to modulate signal transduction and body weight gain through inhibition of protein phosphorylation [58].

Recently, an additional report corroborated that intracellular peptides could modulate obesity and diabetes. Li et al. evaluated the primary cells of brown adipose tissue, and among the altered intracellular peptides, four distinct precursor protein derivatives were identified that play a role in elevating the expression of proliferator-activated receptor-γ coactivator (PGC1-α) and UCP1. Metformin, a classic drug in the treatment of type 2 diabetes, drastically changes the profile of intracellular peptides from the primary culture of human visceral adipocytes. In addition, four peptides derived from the ATP binding cassette subfamily A member 1 (ABCA1), apolipoprotein B (APOB), and cAMP response element-binding protein (CREB) showed potential in the treatment of obesity [59]; however, they were not pharmacologically analyzed. These results corroborate a promising association between intracellular peptides and adipocyte metabolism [57,58].

Intracellular peptides are mainly generated by proteasome [55,56,60,61,62,63] and should function inside the cells, possibly regulating protein interactions [43,54,64,65]. However, a significant portion of intracellular peptides can be secreted [66], suggesting that they can also act on membrane receptors [67,68]. Secreted intracellular peptides can be hydrolyzed by membrane bound or secreted peptidases, such as angiotensin-converting enzyme, Nln, and/or THOP1 [50,69,70,71,72,73,74].

### 2.1. Therapeutics Pharmacology of Intracellular Peptides HP and Pep19 to Ameliorate Overweight, Obesity, and T2D

The potential therapeutic application of intracellular peptides was investigated. Parallel to the identification of intracellular peptides, our group developed a powerful tool, conformational-sensitive antibodies, to identify intracellular peptides targeting GPCR [75,76,77,78,79]; GPCR are well known as major drug targets for treating human diseases [80,81]. Using conformational-sensitive antibodies, novel peptide-based molecules, such as HP and DIIADDEPLT (Pep19), which target cannabinoid receptors, were identified [52,67,79]. HP, HP-containing peptides, and Pep19 potential therapeutic applications for treating overweight, obesity, and T2D will be reviewed below.

#### 2.1.1. HP and HP-Containing Peptides Therapeutics Pharmacology Characterization

HP was the first intracellular peptide identified and pharmacologically characterized as subtly decreasing blood pressure in anesthetized rats [79]. HP is a nine-residue peptide derived from the α-chain of rat hemoglobin, which was first identified in rat brain extracts using the THOP1 substrate-capture assay [44] and was later shown to have inverse agonist pharmacological activity on CB1R [79]. HP is the prototype of a whole new family of peptide-based endocannabinoids (i.e., RVD-HP and VD-HP; and, VDPENFRLLGNM, VD-βHP). However, the endogenous nature of HP remains elusive [75,76,79]. HP’s peptide core is highly conserved among species, and it is naturally produced in mice as RVD-HP (also denominated pepcan12) or VD-HP (also denominated pepcan11) [82,83]. HP is an inverse agonist of type1-cannabinoid receptor (CB1R), and RVD-HP and VD-HP are characterized either as CB1R agonists [76] or negative allosteric modulators [83]. HP and HP-derived peptides support a contemporary view that endocannabinoids also exist as non-lipid-derived products [76,79,83,84,85]. HP demonstrates antinociceptive and anorexigenic properties [79,86]. Acting as an inverse agonist of CB1R, HP displays its antinociceptive property when administered by either intrathecal, intraplantar, or oral routes, underscoring HP’s therapeutic potentials [86]. Further immunohistochemistry studies showed that HP activates key feeding-related brain nuclei of the mediobasal hypothalamus and descending pain pathways of the periaqueductal grey (PAG) and dorsal raphe, but not the higher limbic structures [67,85]. Thus, HP may have selective behavioral effects on nociception and appetite, without engaging reward pathways [68,85,87,88]. A recent study examining the effect of peripheral administration of HP, either intraperitoneal or oral, reported a reduction in food intake in rats and mice [84]. HP dose-dependently decreased night-time food intake in normal male rats and mice as well as in obese ob/ob male mice when administered centrally or systemically, without causing any obvious adverse side effects. The anorectic effect of HP is absent in CB1R null mutant male mice [84].

HP and HP-containing peptides are able to modulate the constitutive activity of CB1R to the same extent as lipid-derived endocannabinoids [44,52,79]. The reason nature built two chemically different class of endocannabinoids remains speculative. One possibility is that the distinctive solubility of hydrophobic lipid-derived endocannabinoids compared to hydrophilic peptide-based endocannabinoids contributes to the increase in the spectrum of endocannabinoids action sites at cells.

To act on plasma membrane receptors, intracellular peptides need to be secreted, which was suggested to occur though a non-classical secretory pathway because intracellular peptide precursors are formed without a signal-peptide sequence to target entry into the secretory pathway [66,68]. HP has a possible limitation for clinical use related to CB1R antagonists, as it is able to induce anxiety similarly to rimonabant [67,89]. Rimonabant was used in the treatment of obesity for a few years; however, it has been removed from the market since 2008 due to its relationship to several cases of depression and suicide.

#### 2.1.2. Pep19 Therapeutics Pharmacology Characterization

Pep19 is a novel and exciting alternative for treating obesity and related metabolic disorders [67]. Pep19 was also identified using conformational-sensitive antibodies as a tool to identify and characterize novel intracellular peptides targeting cannabinoid receptors. Pep19 is an exciting molecule for treating overweight, obesity, and metabolic disorders because it is safe regarding central nervous effects [67]. Pep19 cannot induce anxiety or depression-like symptoms, nor can it induce cannabinoid tetrad behavior or activate cerebral areas related to CB1R activation [67]. Pep19 has the positive clinical benefits of cannabinoids, improving metabolic parameters of obesity both in vivo and in vitro. Pep19 was obtained after rational modifications of a natural intracellular peptide from peptidyl-prolyl *cis-trans* isomerase A (DITADDEPLT) [90]. Pep19 was designed to have increased inverse agonist activity on CB1R without having the undesired central nervous system (CNS) side effects of the previous cannabinoids. These characteristics confer the therapeutic advantages of Pep19 in relation to HP and rimonabant [67].

Currently, CB1R antagonists and inverse agonists can favor weight loss through peripheral actions on the CB1R of adipose cells, increasing metabolism (i.e., inducing browning) [91,92,93]. *Browning* represents the modification of white adipocytes (that store energy) into brown adipocytes (that are related to non-shivering thermogenesis). Brown adipocytes express high levels of uncoupling protein 1 (UCP1), a protein that, when stimulated, dissipates protons from ATP synthesis to heat production [94]. Thus, during browning, white adipocytes start expressing UCP1, which is also present in adult humans [95]. Pep19 increased UCP1 expression in inguinal adipose tissue of obese rats and in 3T3-L1 adipocytes, indicating a peripherical mechanism that involves browning induction, followed by weight loss and improvement of metabolic parameters. The pharmacological effects of Pep19 increasing UCP1 levels in 3T3-L1 adipocytes was blocked by the CB1R antagonist AM251, further implicating CB1R’s activity of Pep19 [67]. However, the cross-pharmacological action of Pep19 on additional targets, such as the β-adrenergic receptors, cannot be excluded as it could also contribute to some of the Pep19 biological effects (Heimann, A.S., personal communication).

Chronic oral Pep19 treatment decreased the body weight of animals with obesity and improved several other metabolic parameters without undesired side effects [67]. When rats with obesity were chronically treated with Pep19, several other metabolic parameters, such as glycemia, serum cholesterol, and triglycerides, improved. A reduction in the adiposity index and the size of white adipocytes in the inguinal tissue of the Pep19-treated animals was observed. Heartbeats were not altered, and blood pressure was reduced in obese animals treated with Pep19. These results showed the pharmacological possibility of using Pep19 to control overweight and obesity, and to improve metabolic parameters such as glycemia and blood pressure. Altogether, these results suggest Pep19 is a promising new peptide candidate for overweight and obesity control and prevention [67]. A mechanism, yet unknown, allows the oral activity of Pep19; similarly, HP and milk protein casein-derived tripeptides IPP (Ile-Pro-Pro) and VPP (Val-Pro-Pro) also have oral activity [96,97,98,99]. However, not much is known yet about the pharmacokinetics of orally active peptides, which may reach the bloodstream and adipocytes tissues without further degradation in the digestive tract.

## 3. Molecular Physiology of Energy Expenditure

The energy balance of the body is controlled by multiple physiological mechanisms that coordinate changes between intake and energy expenditure, regulating body weight around a set point that provides the required energy to maintain homeostasis in the body [100,101]. The mechanisms regulating food ingestion integrate different neural and endocrine signaling systems [102].

In most cases, the neural and endocrine signaling molecules are peptides that bind to specific plasma receptors belonging to either G-protein coupled or tyrosine kinase receptors families. Orexigenic and anorexigenic signaling peptides are responsible for regulating the onset of hunger and satiety [103,104]. Orexigenic or anorexigenic, neural, and endocrine signaling peptides bind to specific receptors that transduce a message into cells to control cellular functioning. Because multiple signaling peptides and receiving receptors exist simultaneously, the signal transduction needs to be integrated within cells to produce a cellular response. The efficacy of the signal transduction depends on factors such as concentration and availability time of the signaling molecules. Therefore, to use the energy stored from food ingestion, the body is prepared with the help of a group of signaling molecules.

The hypothalamus links the nervous and endocrine systems through the anterior pituitary gland and is a key region in the brain to regulate food intake, energy balance, energy storage, body temperature, thirst, and water intake, sleep and wake patterns, reproduction, and growth [105,106,107]. Peptides with orexigenic and anorexigenic properties reach the hypothalamus after being released by brain neurons or coming from endocrine circulation [48]. Orexigenic peptides, such as ghrelin (produced by stomach) and neuropeptide Y or agouti-related peptide (NPY or AgRP) produced by hypothalamic neurons), have receptors in the hypothalamus and promote hunger [108,109]. Anorexigenic peptides from the endocrine system including leptin (produced by white adipose tissue), peptide tyrosine tyrosine 3–36 (PYY 3–36; produced by the gut), islet amyloid polypeptide amylin, pancreatic polypeptide (PP, produced by the pancreas), and ciliary neurotrophic factor (CNTF, produced by skeletal muscle) reach the hypothalamus through blood circulation promoting and maintaining satiety after and between meals [108].

Endocannabinoids derived from biological membranes phospholipids (EDMP) and the cannabinoid type 1 receptor (CB1R) are produced and expressed in the hypothalamus [110,111]. EDMP levels increase significantly within the hypothalamus in response to fasting, and return to normal after eating again [112]. EDMP locally regulate appetite and food intake by modulating the activity of hypothalamic neurons and, subsequently, the release of orexigenic and anorexigenic neuropeptides, as well as the function of mesolimbic and brainstem neurons, thereby participating in both the homeostatic (i.e., based on energy balance) and hedonic (i.e., based on the incentive value of food) aspects of food intake. EDMP are not confined to the CNS and data obtained using pharmacological and genetic tools inactivating CB1 receptors point to the inhibition of sympathetic inputs upon brown adipose tissue (BAT). Also, decreased thermogenesis is one of the most important mechanisms through which CB1R activation reduces energy expenditure by BAT [91] and causes white adipose tissue accumulation in diet-induced obesity (DIO) mice [113,114].

Since the hypothalamus controls eating behavior, its interference with food intake commonly causes complex behavioral alterations in patients, including depression [115,116,117,118]. Not only peptides but also exogenous and endogenous cannabinoid agonists are known to stimulate food intake, and the specific cannabinoid type 1 receptor (CB1R) antagonist/inverse agonist SR141716 (also known as rimonabant) reduces food intake [119,120]. Treatment with the anorexigenic leptin peptide decreases EDMP levels in normal rats and obese (ob/ob) mice, indicating that molecules with a distinct chemical nature (i.e., peptides and plasma membrane phospholipid-derived compounds) can control food intake and energy balance [120]. Together, these findings provide evidence of the role of hypothalamic peptides and EDMP in food intake and appetite regulation [121].

Therefore, the EDMP system is as an attractive target for the development of anti-obesity drugs [122], especially since smoking marijuana has been found to lead to an increased desire to eat (called the “munchies”), presumably by activating the hypothalamic CB1R [123,124]. A significant effort was directed toward developing a select antagonist that would block the receptor’s activity and decrease food intake. This led to the identification of rimonabant, which was found to be a highly selective antagonist (with inverse agonist properties) and reduced feeding and body weight in a number of rodent models [125,126,127]. However, its use as an anti-obesity drug is limited due to its severe central side effect of depression and increased risk of suicide [128,129]. Therefore, the possibility of developing novel molecules targeting CB1R that do not cross the blood brain barrier but still produce the beneficial effects of reducing body weight and improving lipid metabolism has become a challenge for academic laboratories and pharmaceutical companies. As mentioned above, Pep19 is an exciting novel CB1R-targeting molecule to treat overweight, obesity, and metabolic disorders because it does not induce anxiety or depression-like symptoms, nor can it induce cannabinoid tetrad behavior or activate cerebral areas related to CB1R activation [67]. These characteristics confer the therapeutic advantages of Pep19 in previously described cannabinoids [67].

## 4. Neural and Endocrine Peptides Regulating Overweight, Obesity, and Energy Homeostasis

The current therapies that reduce or prevent body weight gain include sibutramine, fluoxetine to control food intake and improve fat metabolism, as well as orlistat, which reduces intestinal fat absorption by inhibiting pancreatic lipases [130]. Currently, none of the existing treatments alone or in combination can normalize blood glucose concentration and prevent debilitating complications from obesity. New types of obesity treatments and safer and more effective antidiabetic agents are needed. New forms of treatments being studied include peptide hormones and their receptors. The use of peptides as actual or potential agents for T2D treatment and anti-obesity will evolve over the years to trigger specific biochemical effects on specific target cells. Thus, using peptides over many potential small molecule drugs, which are xenobiotic and can lead to nonspecific effects and generate toxicity, has many advantages [131].

A major focus of these new therapies is peptide hormones, including glucagon-like peptide 1 (GLP-1), glucose-dependent insulinotropic polypeptide (GIP), cholecystokinin (CCK), and peptide YY (PYY) [132]. They act as hormonal signals that link food absorption to physiological responses such as secretion and insulin satiety. For the treatment of type 2 diabetes, GLP-1 mimetics and GLP-1 degradation inhibitors (DPP4 inhibitors) are many, and GLP-1 mimetics are also licensed [133]. These drugs can be used alone or in combinations. For example, a rationally designed monomeric peptide can reduce body weight and diabetic complications in rodent obesity models by acting on GLP-1, GIP, and glucagon receptors [134]. Regular physical activity needs to be associated with pharmacological control of overweight and obesity. However, despite these well-known possibilities for treatment and prevention, obesity is rapidly becoming an epidemic in developed countries. The currently available anti-obesity therapies are only modestly effective and have significant adverse effects [135].

**Figure 1:**
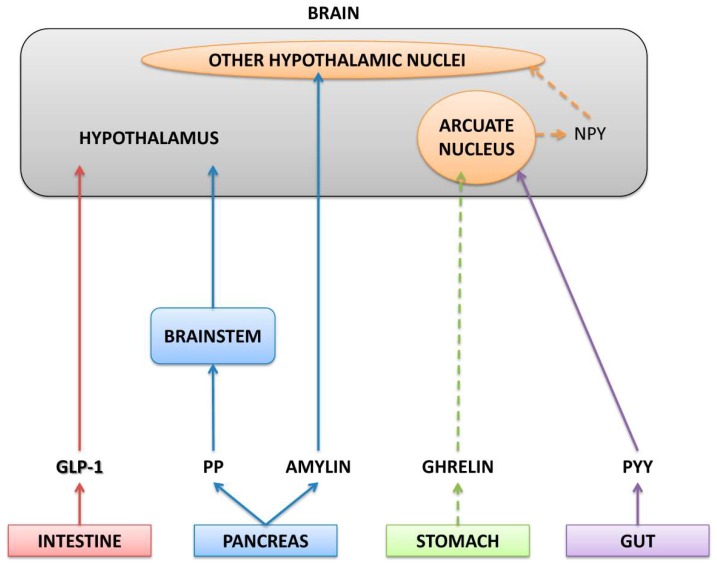
Schematic diagram illustrating neuropeptides. Continuous lines represent anorexigenic peptides that promote satiety; discontinuous lines represent orexigenic peptides that promote hunger. NPY: neuropeptide Y; PYY: peptide YY; PP: pancreatic polypeptide; GLP-1: glucagon-like 1.

Neuropeptides and hormonal peptides synthesized and released by the central nervous system (CNS) and endocrine system are also important for regulating energy homeostasis. The hypothalamus is the brain region that regulates energy homeostasis, and some peripheral peptide hormones acting via the vagus nerve can influence energy homeostasis in the hypothalamus. These peptide hormones are produced in peripheral locations throughout the body, such as the gastrointestinal tract and the pancreas. Therefore, together, hypothalamic neuropeptides and peripheral peptide hormones regulate energy homeostasis and food intake in mammals. Some of these neuropeptides and hormonal peptides and their roles in energy balance are discussed below (Figure 1).

### 4.1. Neuropeptide Y

Neuropeptide Y (NPY) is composed of 36 amino acids. It is a neuropeptide playing important roles in the control of energetic homeostasis. NPY is part of the PP-fold peptide family, which also has the YY (PYY; composed of 36 amino acids) hormone peptide and the pancreatic polypeptide (PP; composed of 36 amino acids) as members. This family of peptides acts through Y receptor signaling. When animals are fasted, NPY expression in the hypothalamus increases, and with a central administration of NPY, it can increase food intake by acting on Y1 and Y5 receptors. NPY is produced in several hypothalamic nuclei; the NPY-containing neuronal population in the hypothalamic arcuate nucleus (ARC) is the best characterized. In this region, NPY is colocalized with agouti-related peptide (AgRP). Both are [136] orexigenic neuropeptides and are considered important sensors of the state of energy [137,138,139]. When activated, by ghrelin for example, they can stimulate feeding. However, they may be inhibited by insulin and leptin [140,141]. NPY is expressed in ARC neurons and can be projected to other different nuclei, such as the paraventricular nucleus, dorsomedial nucleus (DMH), and lateral hypothalamic area [142,143]. One of the functions of ARC’s NPY is to act as a mediator downstream of leptin and thus maintain energy homeostasis [144]. For the dorsomedial hypothalamus (DMH) region, a NPY knock-down study in DMH resulted in development of brown adipocytes in inguinal white adipose tissue, increased interstitial brown adipose tissue (BAT) activity, and expenditure of body energy in addition to feeding [145].

### 4.2. Peptide YY

Peptide YY (PYY) is an anorexigenic hormone composed of 36 amino acids. It is expressed in the gastrointestinal tract by enteroendocrine L cells, with highest concentrations in the terminal ileum, colon, and rectum. PYY can be expressed in two main endogenous forms: PYY 1–36 and PYY 3–36 [146]. Upon release, PYY 1–36 undergoes the action of dipeptidyl peptidase-IV (DPP-IV) and is converted to PYY 3–36. Both forms of PYY bind to the Y2 receptor. However, PYY 1–36 can also bind to two other receptors subtypes (Y1 ad Y5). PYY 3–36 is the main circulating form. Its action via Y2 modulates the neuronal activity of the hypothalamic arcuate nucleus, consequently reducing food intake in mice and humans [147,148]. To be released from the gut into the circulation, PYY depends on the nutrients. Some factors that influence the level of PYY peak include caloric load, macronutrient composition, and feed consistency. During fasting, the PYY levels are lower [149,150]. Studies showed that rats fed a high-fat diet have deficient PYY secretion [147,151]. In PYY 1–36 analysis, a reduction in food intake in rodents was also observed after a peripheral infusion, although with a lesser potency compared to PYY 3–36 [152]. These data showed that PYY analogs act as regulators of body weight. Thus, interest in using them as a therapy for the treatment of obesity is strong.

PYY is found in the pancreas [153] and its relationship with diabetes was studied. Studies showed that PYY inhibits glucose-stimulated insulin secretion (GSIS) from islets isolated from mice, which may be a result of β cell resting induction. In mice, selective elimination of PYY-expressing cells results in hyperglycemia as a result of increased β cell destruction rather than decreased secretory function [154]. Previous studies demonstrated the relationship of Y1 receptors in these responses. Y1 receptor-specific knockout mice revealed hyperinsulinemia and increased pancreatic insulin stores, leading to the onset of obesity [155]. More recent work indicated a role for PYY receptor activation by PYY in modulating β cell function and maintenance [156]. The Y2 receptor-specific agonist PYY 3–36 [157] was shown to cause insulinostatic effects similar to those of native PYY [156]. PYY was also studied as a key factor in the remission of diabetes after bariatric surgery in rats. The data showed that patients after bariatric surgery have high levels of PYY, which may be associated with an improved glucose response [158]. Currently, drugs based on combination therapy and multi-agonism are being developed and studied in clinical trials to provide a new generation of therapies for diabetes and obesity. PYY analogs combined with glucagon-like peptide-1 (GLP-1) agonists are currently under development for the treatment of obesity [159]. Studies showed that co-administration of PYY3–36 with GLP-17–36 amide was more effective in inhibiting appetite than any of the isolated peptides [160]. This combination also reduced energy intake compared to the placebo and more than the sum of individual infusions, demonstrating a synergistic effect [161]. Another example of combined therapy is triple agonism, with unimolecular agonists using GLP-1/oxyntomodulin/PYY [159]. One study showed that subcutaneous infusion of the hormones GLP-1, OXM, and PYY replicated the postprandial levels of intestinal hormones observed after Roux-en-Y gastric bypass surgery (RYGB), which led to improved glycemic control as well as weight loss, with other beneficial metabolic effects [162,163]. In addition to glucose and insulin levels after main meals, they were significantly lower in the infusion of GLP-1, OXM, and PYY groups compared to the placebo [163].

### 4.3. Pancreatic Polypeptide (PP)

Pancreatic polypeptide (PP) is a peptide of 36 amino acids. It is produced by specialized pancreatic islet cells, called F cells, through a vagal cholinergic mechanism after a meal. The intake of nutrients, especially lipids, stimulates the release of PP, which has anorexigenic effects and can act to reduce food intake. It can also influence the energy balance and body composition, playing an important role in the regulation of energy homeostasis [164,165,166]. Dose-dependent PP in fed and fasted animals causes a decrease in food intake. This action is mediated via the Y4 receptor. This receptor is expressed in the hypothalamus and brain stem, notably in the postrema area and dorsal nucleus of the vagus nerve [167]. An experiment with Y4 mice produced the loss of the anorectic effect of PP, confirming that the peptide acts through the Y4 receptor [168]. Studies showed the interaction of PP with other members of the NPY family, such as PYY. Together, they were shown to aid in the delay of gastric emptying and the reduction of food intake [169]. Healthy subjects who received a peripheral infusion of PP had significantly decreased food intake caused by a reduced appetite, delayed gastric emptying, and gastric attenuation [170,171].

PP also plays an important role in glucose homeostasis, decreasing insulin resistance by hepatic glucose production reduction. The hormone released by PP cells inhibits insulin secretion in the body, and PP cell percentage and distribution increase significantly in the course of diabetes [172]. A study of individuals with diabetes showed that 12 weeks of calorie-restriction-induced weight loss improved β cell function, insulin resistance, and the role of gastrointestinal hormones in T2D. In these subjects, a decrease in plasma PP concentrations was observed after diet-induced weight loss and a consequent decrease in fasting and stimulated insulinemia. The data suggested that PP may play an important role in mediating the improvement of β cell function, and, combined with dietary and exercise interventions, provide an effective means for positively regulating intestinal peptide function in individuals with T2D [173].

### 4.4. Glucagon-Like 1 (GLP-1)

GLP-1 is a peptide hormone of 31 amino acids. It is synthesized and secreted into the intestine from enteroendocrine L cells [174]. GLP-1 can be synthesized by pre-proglucagon (PPG) neurons in the brain [175]. GLP-1 secretion is stimulated by the meal ingested. The presence of nutrients in the lumen of the intestine favor its secretion, as well as neural and/or endocrine mechanisms, which are also apt to operate. The opposite is observed in the fasted state, where GLP-1 concentrations are very low [176,177,178]. By inhibiting gastric emptying, GLP-1 reduces plasma glucose levels and body weight. Consequently, food intake decreases, thus reducing postprandial glucagon secretion. GLP-1 also is a potent glucose-dependent insulin stimulant [174,177]. The physiological effects of GLP-1 were explored for the development of treatments to alleviate hyperglycemia and reduce excess weight. One study showed that activation of the GLP-1 receptor reduces food reward and, thus, decreases the craving for food [179]. In recent years, new therapeutic strategies based on the GLP-1 system were introduced into clinical practice for the treatment of T2D. Available medications include long-acting injectable GLP-1R agonists and dipeptidyl peptidase-4 (DPP-4) inhibitors that increase the bioavailability of endogenous GLP-1 [180]. DPP-4 regulates circulating active GLP-1 levels and also acts on other peptides involved in metabolic regulation, including glucose-dependent insulinotropic polypeptide (GIP) [181,182]. Partial inhibition of DPP-4 can increase the circulating half-life of GLP-1 and GIP by two- to three-fold. A study in mice suggested that increased bioavailabilities of GIP and GLP-1 contribute to the ability of DPP-4 inhibitors to improve glucose tolerance [183].

Liraglutide is an approved GLP-1 agonist, approved by some countries for weight management and treatment of T2D. In 2010, the Food and Drug Administration (FDA) approved daily subcutaneous injection of 1.8 mg of liraglutide as an adjuvant to dietary and exercise therapy for the treatment of T2D [184]. A recent scientific study showed that liraglutide provides better glycemic control without major damage to renal function or increased risk of hypoglycemia [185]. That same year, the FDA approved a daily subcutaneous injection of 3.0 mg of liraglutide for chronic weight control in obesity and/or overweight patients [186]. It is used as a complement to a hypocaloric diet and increased physical activity. Some studies showed that subjects treated with 3.0 mg liraglutide experienced a dose-dependent weight loss of about 6 to 8 kg. Meanwhile, placebo-treated individuals who applied only one of diet or exercise without the use of the drug experienced an average weight loss of between 0.2 to 3.0 kg [187,188,189]. The researchers also observed that in a study after 56 weeks, 3.0 mg liraglutide was able to improve glucose metabolism by reducing the progression of type 2 diabetes in overweight or obesity subjects with pre-diabetes [190].

### 4.5. Ghrelin

Ghrelin is an acylated peptide composed of 28 amino acids [191]. It is an orexigenic hormone and was first discovered as the endogenous ligand for the growth hormone secretagogue receptor (GHS-R). It is expressed in the hypothalamic ARC and periventricular area, and exerts its action through its association with specific hypothalamic receptors [192]. Ghrelin is synthesized in the stomach, being secreted mainly by the endocrine cells of the oxyntic glands of the gastric fundus. It is also synthesized in the body of the stomach, mucosa of the duodenum and jejunum, and by the lungs, urogenital organs, and the pituitary gland, but to a lesser extent [193]. Its diverse actions in the human body range from its involvement in the regulation of the immune and cardiovascular systems to the positive regulation of insulin-like growth factor. Its dominant role in the gastrointestinal system involves gastric emptying and intestinal motility [194,195]. The actions of ghrelin are implicated in different physiological processes, such as its association with food intake, adiposity, and regulation of metabolism. Thus, ghrelin has become the focus of research and is considered a target for the treatment of obesity.

Ghrelin levels rise before meals and fall postprandially [196]. Some studies showed that different forms of acute (central or peripheral) administration of ghrelin in rodents stimulate feeding [192]. Chronic administration also alters homeostasis by inducing weight gain [197]. A phase II clinical trial showed that patients who had a severe reduction in body weight for more than one year after using gastrectomy while receiving treatment with synthetic human ghrelin had improved food intake. With this, the treatment of synthetic ghrelin proved to be effective in the treatment of loss of appetite and weight loss. However, ghrelin resistance in obesity is still unclear. Resistance to ghrelin was proposed as a mechanism to protect against overweight.

The effect of ghrelin on glucose metabolism regulation has gained increasing recognition because pharmacological inhibition of ghrelin signaling might important in the treatment of insulin resistance in type 2 diabetes. Many studies on a variety of species, including mice [180] and humans [185], evaluated the effects of ghrelin on glucose metabolism. Mice deficient in ghrelin or its receptor exposed to a high-fat diet showed better glucose tolerance and insulin sensitivity [187,188] compared to controls. In a study with ob/ob mice, ghrelin deletion decreased hyperglycemia and improved glucose-induced insulin secretion, thus improving insulin sensitivity in peripheral tissues compared with ob/ob controls [189]. However, ghrelin does not only affect glucose metabolism by directly inhibiting the stimulation of insulin secretion by glucose. Ghrelin and its receptor are also produced in pancreatic cells. The blockade of pancreatic ghrelin resulted in increased insulin secretion and decreased high-fat diet (HFD)-induced glucose intolerance in mice. Several lines of evidence suggest a role of ghrelin in affecting glucose metabolism by stimulating α cell glucagon secretion [198,199,200].

Ghrelin also plays a role in fat metabolism and glucose homeostasis. The crosstalk between lipid and glucose metabolism may indicate a physiological role of ghrelin in insulin resistance. Ghrelin is involved in the regulation of metabolic hormones, and GHS-R is present in adipose tissue [192,201], where it has been shown to play a role in adiposity. Reduced ghrelin levels in patients with T2D are associated with increased abdominal adiposity and insulin resistance [201,202]. The association between obesity and T2D is well described; ghrelin signaling pharmacologically functions in the prevention or treatment of this disease. The compound YIL-781 demonstrates a selective affinity for GHS-R1α. It is a competitive antagonist of GHS-R1α and results in the blockade of the ghrelin binding domain. Testing of insulin-resistant DIO rats through oral administration of YIL-781 showed a reduction in fat mass and increased glucose-stimulated insulin secretion [202,203].

### 4.6. Amylin

Amylin, or amyloid islet polypeptide (IAPP), is a peptide hormone of 37 amino acids secreted by pancreatic β cells. It is co-stored and co-secreted with insulin and they act synergistically to control blood glucose levels [204]. Recent data showed that amylin expression also occurs in the CNS. More specifically, expression occurs in parts that act on metabolic control, such as the lateral hypothalamus (LH) [205]. Amylin is a member of the calcitonin family of peptides and activates the calcitonin receptor when dimerized with a receptor activity-modulating protein [206]. Among its functions, amylin controls nutrient fluxes by reducing energy intake, modulating nutrient use, and increasing energy expenditure. However, the role most studied and investigated is that as a sign of satiety [207,208]. These discoveries were important for new findings that would lead to amylin ablation as a treatment for unhealthy weight in obese individuals. Animal experiments suggested that when administered centrally, chronic amylin infusion reduced body weight in rats regardless of initial body weight and acute energy expenditure [209].

The clinical utility of amylin is restricted by its instability and its tendency to self-aggregate. Current studies showed that the pathophysiology of diabetes is not only associated with changes in insulin secretion, but also with abnormal amylin regulation. Patients with type 2 diabetes who use insulin also have a decreased amylin response to caloric intake, potentially related to the degree of β cell impairment [210]. In the pancreas, the native form aggregates to form amyloid fibrils, generating a cytotoxic aggregate that may contribute to pancreatic β cell dysfunction in diabetes [211,212]. Many diabetic patients have an amylin deficiency, and a synthetic analog of amylin was developed to support their treatment. This new compound is soluble and non-aggregating and is currently approved for use with insulin by type 1 and 2 diabetics. Over the course of a year, the treatment of patients with type 1 and 2 diabetes resulted in sustained weight loss. Thus, this analog is currently being tested in non-diabetics as a treatment for obesity [213,214]. Another study suggested that amylin can be used in combination with leptin as a weight loss therapy [215,216].

## 5. Food-Derived Bioactive Peptides in Obesity and Related Metabolic Disturbances

Food proteins are an important source of energy and essential amino acids for normal growth, life maintenance, and reproduction. In addition to their nutritional value, their partial digestion by proteases may produce peptide sequences with specific biological properties (bioactive peptides), acting as physiological modulators both locally in the gut and systemically [217]. The discovery of food-derived bioactive peptides opened a whole new perspective on protein nutrition and the role of proteins and protein hydrolysates in metabolism and health [218].

These biologically active peptides were identified and isolated from animal and vegetal sources. Bovine milk, cheese, and dairy products are the largest sources of bioactive peptides derived from food [219,220]. However, they can be obtained from other animal sources, such as bovine blood, meat, eggs, and fish. Wheat, maize, soy, and rice are also sources of bioactive peptides [221].

Bioactive peptides are predominantly encrypted or inactive within food protein and become active once they are released from their precursor protein [219,222]. In vivo, encrypted peptides can be liberated during gastrointestinal digestion by enzymes such as trypsin or by microbial enzymes. In vitro, bioactive peptides can be released during food processing or ripening by microbial enzymes (e.g., *Lactobacillus helveticus*) [223,224,225,226]. These peptides are short sequences of approximately 2–20 amino acids [227] and, to be transported intact to the target site or organ, bioactive peptides must escape degradation during digestion. Different transport systems for the intestinal absorption of peptides were described. Smaller peptides are transported by a specific peptide transporter (PepT1) [228,229] located in the brush border membrane. Oligopeptides can be transported by transcytosis (vesicle-mediated transcellular transport) and paracellular pathways [229]. The paracellular pathway is a non-degradative transport route and is suggested to be the main mechanism for transport of intact peptides. The hypothesis that some small peptides escape degradation and are transported from the intestinal lumen into circulation is gaining acceptance, and an increasing number of in vitro studies describe the transepithelial transport of bioactive peptides [230,231]. The molecular size, weight distribution, and other properties of peptides, such as hydrophobicity, can determine the major transport route for peptides [232].

**Figure 2:**
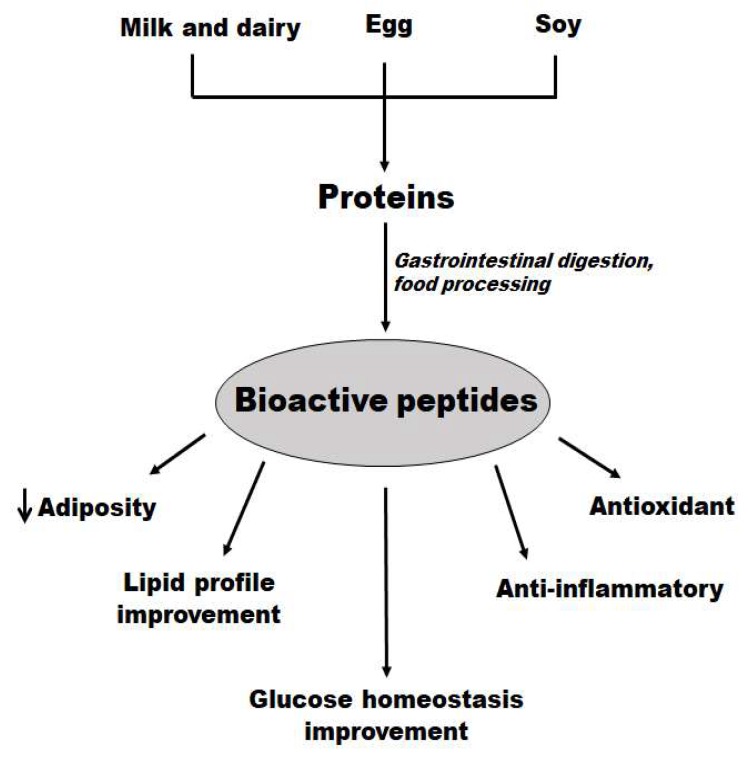
Summary of the main effects of bioactive peptides from food on obesity and related metabolic disturbances.

Their diversified structures create the several functions of food-derived bioactive peptides, including an important anti-obesogenic potential by modulating several metabolic disturbances associated with obesity, such as insulin resistance, inflammation, oxidative stress, and dyslipidemia. Here, we review the main food-derived bioactive peptides with effects related to preventing and/or treating obesity and its metabolic alterations. We review peptides from milk, egg, and soy. Peptides from legumes [233], vegetables [234], and meat [235,236] were recently reviewed elsewhere (Figure 2).

### 5.1. Milk-Derived Bioactive Peptides

Dairy foods are an important part of the Western diet as a source of good quality protein and several vitamins and minerals. The consumption of dairy foods was suggested to be beneficial in the regulation of body weight [237,238], as well as metabolic parameters [239]. Among other animal-derived food sources, milk proteins are considered a highly nutritious food component with a well-balanced essential amino acid composition and were reported as a good source of bioactive components [240,241,242].

Milk protein is comprised primarily of whey and casein proteins, which constitute about 20% and 80% of the total protein fraction, respectively [243]. The whey proteins include β-lactoglobulin, α-lactalbumin, serum albumin, immunoglobulins, lactoferrin, and lactoperoxidase, in addition to other minor proteinaceous components [244]. The gastrointestinal digestion of whey protein leads to generation of many bioactive peptides and amino acids [229,245], which stimulate several gut hormones associated with food intake regulation, such as cholecystokinin (CCK), PYY, and ghrelin [246,247,248,249]. Whey protein was shown to increase the release of CCK and PYY and reduce ghrelin secretion, underlying a potential role in hunger suppression via reduced food intake and increased satiety in men and woman with obesity [250,251].

Some experimental and clinical studies suggest that exogenous lactoferrin administration may be a promising pharmaceutical agent to reduce fat accumulation. In DIO rats, lactoferrin produced sustained weight and fat loss, and attenuated the reduction in thermogenesis associated with calorie restriction [252]. Dietary consumption of lactoferrin during caloric restriction in mice improved weight loss and induced a strong decrease in adiposity and adipocyte size [253]. Similarly, bovine lactoferrin administration to mice reduced mesenteric fat mass but failed to modulate body weight [254]. In human subjects, treatment with enteric-coated lactoferrin tablets for eight weeks reduced visceral fat in men and women without the need for any lifestyle change [255]. This result may be due to the promotion of lipolysis and the additional anti-adipogenic activity of lactoferrin [256]. Bovine lactoferrin was found to produce beneficial effects on plasma lipid concentrations. Its administration in rodents led to increased plasma high-density lipoprotein (HDL) cholesterol concentrations, decreased plasma concentrations of triacylglycerol and non-esterified fatty acids, and decreased hepatic cholesterol and triacylglycerol concentrations [257,258,259,260,261,262]. Lactoferrin is also produced by neutrophils, and some human studies showed its plasma levels are negatively associated with overall adiposity and an altered glucose tolerance [263,264]. In addition, the circulating lactoferrin concentration and lactoferrin polymorphisms were linked to the plasma lipid profile [265]. Decreased circulating lactoferrin was associated with insulin resistance and type 2 diabetes [263].

Another important milk-derived whey protein with therapeutic potential against obesity and obesity-related disorders is α-lactalbumin [253,266,267]. α-lactalbumin exhibits different anti-obesity effects such as hunger suppression, decreased weight and fat gain, increased thermogenesis and protein balance, and decreased fat balance [252,267]. Similar to lactoferrin, lactalbumin is also able to decrease plasma leptin and insulin, as well as increase peptide YY [252].

Peptides derived from casein hydrolysis show satiety effects through different mechanisms such as the stimulation of CCK release [268] and glucagon-like peptide 1 (GLP-1) release. GLP-1 plays a significant role in energy homeostasis: it regulates blood glucose via its incretin action and promotes satiety and food intake decrease via its anorexigenic properties. Additionally, casein-derived hydrolysates were shown to stimulate the 5-HT2c serotonin receptor, suggesting a potential appetite-suppressing effect [269].

### 5.2. Egg-Derived Bioactive Peptides

Eggs are relatively cheap, found in almost every country, and are nutrient-dense, which means they could be affordable and beneficial to a broad range of the world’s population [270]. In addition to the nutritional value, egg proteins are a source of peptides with a myriad of bioactive properties [271].

In vitro studies demonstrated a remarkable antioxidant activity of egg white protein hydrolysates [272,273,274]. In 3T3-F442A cells, egg white hydrolysate promoted adipocyte differentiation through a combination of insulin mimetic and insulin sensitizing actions. This hydrolysate induced the expression of the anti-inflammatory hormone adiponectin and suppressed the cytokine-mediated inflammatory response in these cells [275]. The authors suggested that bioactive peptides in the egg white hydrolysates are potentially responsible for the observed effects.

To evaluate the potential of egg white hydrolysates in vivo, obese Zucker rats received pepsin egg white hydrolysate or *Rhizopus* aminopeptidase egg white hydrolysate for 12 weeks [276]. The consumption of egg white hydrolyzed with pepsin significantly decreased the epididymal adipose tissue, improved hepatic steatosis, and lowered plasmatic concentration of free fatty acids in the obese animals. It also decreased plasma levels of tumor necrosis factor-α (TNF-α) and reduced oxidative stress.

Recently, the same research group investigated the effect of egg white hydrolysates on glucose metabolism complications related to metabolic syndrome [277]. Hydrolysates with pepsin or with aminopeptidase were administered to obese Zucker rats for 12 weeks through their drinking water. The most promising results were obtained with the hydrolysate of egg white with pepsin, which was able to lower plasma insulin levels and improve insulin sensitivity. The intake of this hydrolysate significantly improved the hepatic steatosis typical of obese Zucker rats. The weight of the epididymal adipose tissue was lower in the animals that received both hydrolysates than in the obesity animals that drank only water. The effect of a pepsin egg white hydrolysate on metabolic complications was also studied in a high-fat/high-dextrose diet-induced metabolic syndrome experimental model [278]. Egg white hydrolysate consumption normalized body weight gain, abdominal obesity, and weight of adipose tissue and liver, and reduced the plasma glucose level. Inflammation and oxidative stress biomarkers were also normalized in supplemented animals. Rats with metabolic syndrome attenuated their body weight gain when they started to consume the hydrolysate, without affecting food intake. Recent data suggest that egg white hydrolysate could directly activate brown adipose tissue metabolism. As a consequence, thermogenesis could be enhanced; consequently, a reduction in the body weight and adiposity could be observed [278].

Obesity and diabetes are two disorders that are associated with microbial dysbiosis and changes in composition and functionality of gut microbiota [279]. The gut microbiota of obesity individuals is proposed to be more efficient at extracting energy from the diet than the microbiota of lean individuals. The weight gain is thought to be explained by several gut-bacteria-related mechanisms, including the microbial fermentation of indigestible dietary polysaccharides into absorbable monosaccharides, and the generation of short-chain fatty acids that are converted to more complex lipids in the liver [280]. Obese Zucker rats supplemented with egg white hydrolysates with pepsin showed a modulation of the microbiological characteristics similar to those of lean rats [281]. Changes in gut microbiota were accompanied by a trend of diminishing fecal short-chain fatty acid levels that occurred simultaneously with a previously reported improvement in markers of oxidative stress and inflammation [276]. Hydrolysate, by virtue of its antioxidant activity and its capacity to reduce inflammation, could modulate the gut microbiota toward a more balanced scenario that lowers short-chain fatty acid production and associated lipogenesis, contributing to reduced fat accumulation and liver steatosis.

### 5.3. Soy-Derived Bioactive Peptides

Soybeans are a rich source of high-quality proteins containing all the essential amino acids found in animal proteins but without cholesterol and less saturated fat [282]. The focus on soy research has shifted to the identification and characterization of bioactive peptides and their corresponding physiological functions.

The best studied bioactivity of soy peptides is their hypolipidemic property [283]. Peptide LPYPR from the glycinin subunit of soybean was one of the initial hypocholesterolemic peptides described [284]. Oral administration of this peptide for two days reduced serum total cholesterol and low-density lipoproteins (LDL) cholesterol in mice. Further studies showed, more specifically, that LPYPR has this hypocholesterolemic effect from acting as a competitive inhibitor of 3-hydroxy-3-methyl-glutaryl-CoA reductase (HMG-CoA reductase), the main rate-limiting enzyme in cholesterol biosynthesis [285], and increasing the ability of HepG2 cells to uptake LDL cholesterol [286]. Other cholesterol-lowering soy peptides were also described [285,287,288,289,290].

Another important physiological function of soy-derived peptides is the antidiabetic effect. A study was conducted to verify the antidiabetic potential of aglycin, a natural bioactive peptide isolated from soybean, in diabetic mice. With the onset of diabetes, the mice were administered aglycin daily (50 mg/kg/day) for four weeks. Treatment with aglycin significantly and effectively controlled hyperglycemia and improved oral glucose tolerance. Aglycin enhanced glucose uptake and glucose transporter recruitment to the C2C12 cell surface in vitro [291].

Regarding the anti-obesity effects, the peptide lunasin was orally administered for 25 weeks to C57BL/6 mice fed a high-fat diet. The treatment was associated with the alleviation of liver damage, reduction in both serum triglyceride and glucose levels, and a decrease in total body fat. Similarly, protease-prepared soy hydrolysate reduced fat accumulation in genetically obese mice, enhanced lipid excretion, and improved plasma cholesterol levels [292]. The reduction in fat accumulation could be due to the higher postprandial energy expenditure observed after intake of protease-prepared soy hydrolysate compared to casein [293]. Although the effect on energy expenditure was not sustained after 24 h, total carbohydrate oxidation continued to be higher in the soy hydrolysate-treated group, perhaps due to higher plasma insulin levels and lower glucose concentrations during the postprandial period or due to lower lipid absorption and increased carbohydrate absorption [293].

An experimental study showed that β-conglycinin, one of the major components of soy protein, is able to suppress food intake and inhibit gastric emptying in rats [282]. These effects were completely abolished by intravenous injection of the selective peripheral CCK receptor antagonist, indicating that endogenous CCK mediates the reduction in food intake by luminal β-conglycinin peptone and that CCK-A receptors are involved in the reduction. The β-conglycinin effectivity in reducing plasma triglyceride and cholesterol levels in rats was also demonstrated [294].

## 6. Undesired and Misuse of Therapeutic Peptides

As mentioned above, peptides function by modulating and modifying the physiological endocrine system, which includes central and peripherical effects. The normal functioning of hormone-producing glands, such as the pancreas, ovaries, testes, thyroid, and others, can be affected by peptides. If peptides are misused to change the physiological functioning of these system, they may have undesired effects. Therefore, peptides also have undesired pharmacological effects similar to structurally unrelated bioactive or pharmacologically active molecules.

Researchers found that children administered synthetic human growth hormone (GH) have a significantly higher risk of developing cancer in the long term [295,296,297,298]. The use of GH to improve performance was also observed and banned from sports [299]. Peptides such as ipamorelin, which has specific GH-releasing properties [300], are powerful medicines and can produce significant adverse effects on the human body, especially when used without medical supervision and at doses outside the recommended range [301]. However, growth hormone-releasing hormone (GHRH) agonist MR409 (N-Me-Tyr^1^, D-Ala^2^, Asn^8^, Arg^29^-NHCH_3_-JI-38) was demonstrated to suppresses tumor growth of human experimental lung and other cancers, including gastric, pancreatic, urothelial, prostatic, mammary, and colorectal [302,303]. Within the heart, GHRH agonists can activate cardiac repair after experimental myocardial infarction in rats, suggesting the existence of a potential signaling pathway based on GHRH in the heart [304], clearly demonstrating that peptides, as with any other pharmacological functional molecules, can have both beneficial and toxic effects depending on the prescription and the dose administrated.

Erythropoietin (EPO) is the main hormone regulating red blood cell (RBC) production, and is another peptide frequently used for sports doping [305,306,307]. In individuals with an iron deficiency, EPO can elevate thrombocyte counts and increase the risk of cardiovascular problems, including cardiac arrest, seizures, arrhythmia, hypertension, congestive heart failure, vascular thrombosis, myocardial infarction, and edema. EPO is also involved in angiogenesis [308], and EPO withdrawal may lead to neocytolysis [305,306,307,309].

## 7. Closing Remarks

Using peptides from natural or rationally designed sources as drugs leads to new and helpful therapies. Peptides, similar to structurally unrelated small molecules, are molecules with high commercial value. For example, the peptide-based medicine Lupron^TM^ (Abbott Laboratories, Park City, IL, USA) for the treatment of prostate cancer and more, achieved global sales of more than USD $2.3 billion in 2011, and Lantus^TM^ (Sanofi, Paris, France), which is on the border between a peptide drug and a small biopharmaceutical, reached sales of USD $7.9 billion in 2013 [310]. The recent identification of intracellular peptides as naturally occurring functional peptides opened a new avenue for the development of novel drugs. Current examples of intracellular peptides with pharmacological applications are HP and Pep19 [52,67]. Pep19, for instance, has several advantages over previously described molecules that target CB1R to treat overweight, obesity, and T2D. In addition, dietary proteins containing encrypted bioactive peptides that function to reduce body weight and improve insulin resistance are potential novel therapeutic possibilities for treating or preventing obesity and associated diseases. Therefore, the peptides presented are viable compounds for overweight, obesity, and type 2 diabetes therapies.
